# Modeling Hematological Diseases and Cancer With Patient-Specific Induced Pluripotent Stem Cells

**DOI:** 10.3389/fimmu.2018.02243

**Published:** 2018-09-28

**Authors:** Huensuk Kim, Christoph Schaniel

**Affiliations:** ^1^Black Family Stem Cell Institute, Icahn School of Medicine at Mount Sinai, New York, NY, United States; ^2^Department of Cell, Developmental and Regenerative Biology, Icahn School of Medicine at Mount Sinai, New York, NY, United States; ^3^Graduate School of Biomedical Sciences, Icahn School of Medicine at Mount Sinai, New York, NY, United States; ^4^Department of Pharmacological Sciences, Icahn School of Medicine at Mount Sinai, New York, NY, United States; ^5^Mount Sinai Institute for Systems Biomedicine, Icahn School of Medicine at Mount Sinai, New York, NY, United States

**Keywords:** cancer, blood disorders, hematopoietic malignancies, induced pluripotent stem cells, model systems

## Abstract

The advent of induced pluripotent stem cells (iPSCs) together with recent advances in genome editing, microphysiological systems, tissue engineering and xenograft models present new opportunities for the investigation of hematological diseases and cancer in a patient-specific context. Here we review the progress in the field and discuss the advantages, limitations, and challenges of iPSC-based malignancy modeling. We will also discuss the use of iPSCs and its derivatives as cellular sources for drug target identification, drug development and evaluation of pharmacological responses.

## Introduction

**Figure 1:**
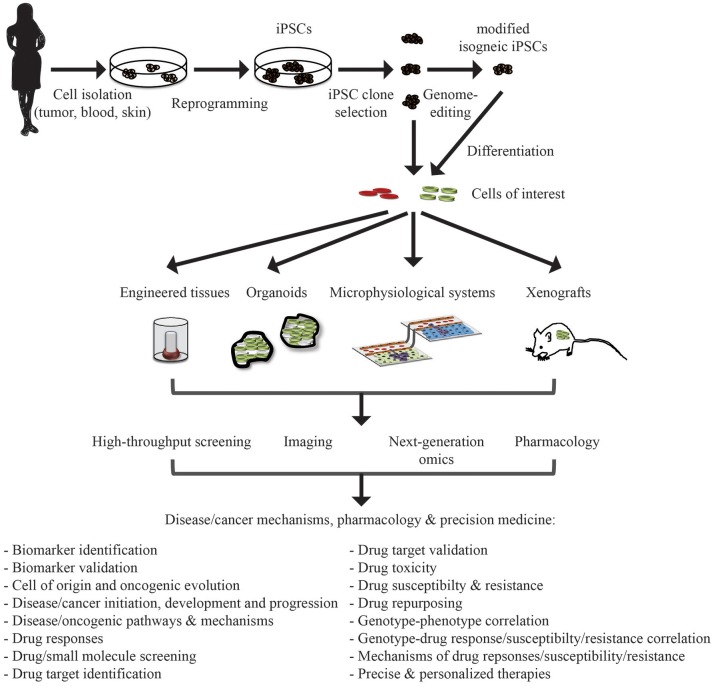
Application of iPSC in disease/cancer modeling, pharmacology, and precision medicine. Patient samples can be collected from a variety of tissue source depending on the need and reprogrammed to iPSCs. These iPSCs can be genome-modified to introduce or correct specific mutations or lesions to generate isogenic iPSC lines for comparative analysis. These iPSC lines can be differentiated into cells of interest (cell type of origin of the malignancy or unrelated cells that may be affected by adverse drug events/toxicity). These differentiated cells can then be integrated, also with other cell types, into engineered tissues, organoids, and microphysiological systems, or xenografted into appropriate *in vivo* model systems. These systems can then be interrogated for understanding disease/cancer mechanisms and signaling pathways, drug discovery and evaluation, and deriving precise personalized therapies.

Hematological diseases and cancers are devastating diseases with a high economic and social burden. Generally basic and preclinical cancer research relies on model systems in order to understand the cellular and molecular mechanisms of the malignant state at the cellular, organ and organism level. The hope is that the information gained from such model systems will be helpful in devising precise, effective, and personalized therapeutic strategies. Prototypically, these model systems include immortalized cell lines and genetically engineered, mutant mice. More recently, advanced patient-derived models such as conditionally reprogrammed cells (CRs) (1–3), patient-derived tumor xenografts (PDXs) (4), CRs combined with PDXs (5), and three-dimensional patient derived organoid cell cultures (6–9), engineered tissues (10–12), and microphysiological systems (MPSs) (13–20) have attracted the interest of the biomedical research community. One particular (r)evolution in modern era biomedical research arose with the breakthrough, Noble-prize awarded discovery of induced pluripotent stem cell (iPSC) generation from somatic cells (21–24). These iPSCs are akin embryonic stem cells, and can be maintained indefinitely in a self-renewing, undifferentiated pluripotent state in culture and be directed to differentiate to any cell type in the body, provided the right cues. Thus, the derivation of iPSCs from patient cells provides a new tool in the arsenal for investigation of disease and cancer pathogenesis, drug development and precision medicine (Figure 1).

## Induced pluripotent stem cell models of hematological diseases, blood cell cancers and non-hematopoietic cancers

The use of iPSCs in the study of hematological diseases, cancer, and tumorigenicity is gaining momentum. It started with the generation of iPSCs from a human melanoma and a human prostate cancer cell line in 2008 (25). Since then numerous malignant cell lines have been reprogrammed that represent among other organs the brain, intestine, liver, lung, pancreas, prostate, and skin, as well as the blood (26–37) (Table 1).

**Table 1:** Human cancer cell line-derived iPSCs.

**Cancer type**	**Cell line reprogrammed**	**References**
Breast cancer	MCF-7	(34)
Cholangiocellular cancer	HuCC-T1	(27)
Chronic myeloid leukemia	KBM-7	(28)
Colorectal cancer	DLD-1, HCT116, HT-29	(27)
Esophageal cancer	TE-10	(27)
Ewing's sarcoma	SK-NEP1, CHLA-10	(30, 36)
Gastric cancer	MKN45	(27)
Glioblastoma multiforme	Glioblastoma multiforme neural stem cell lines G7 & G26	(32)
Hepatocellular cancer	PLC	(27)
Liposarcoma	SW872	(30)
Lung cancer	A549, H358, H460	(29, 31)
Melanoma	Colo, R545	(25, 26)
Oral squamous cell carcinoma	H103, H376	(37)
Osteosarcoma	Saos-2, HOS, MG-63, G-292, U2OS	(30, 35)
Pancreatic cancer	MIAPaCa-2, PANC-1	(27)
Prostate cancer	PC-3	(25)

The reprogramming of cancer cell lines was soon followed by the generation of iPSCs representing various hematological diseases, blood cell cancers, and non-hematopoietic cancers (38–77) (Table 2). These iPSCs were derived from primary patient cells, cancerous tissues or patient cells harboring known oncogenic lesions. In Table 2 we summarize whether functional assays were performed in attempt to phenocopy the disease/malignancy, describe the phenotypes observed and whether the studies used genome editing to either create or correct disease/cancer-associated mutations.

**Table 2:** Current patient-specific iPSC models of hematological diseases and cancer.

**Hematological disease or cancer type**	**Functional assay(s)**	**Disease/Cancer Recapitulation**	**Genome editing**	**References**
8p11 myeloproliferative syndrome (EMS)	*In vitro*	Yes—increased output in granulocyte-erythrocyte-macrophage-megakaryocyte, erythrocyte and macrophage colonies	No	(72)
AML	*In vitro* & *in vivo*	Yes—preferential *in vitro* generation of granulocyte-macrophage, granylocyte and macrophage colonies and aggressive myeloid leukemia *in vivo*	No	(41)
	*In vitro*	Yes—reduction in blood cell specification and block in generation of granulocyte-macrophage and erythroid colonies	No	(59)
Aplastic anemia	*In vitro*	Yes—impaired proliferation of hematopoietic progenitors and reduced erythrocyte and myeloid cell output -	No	(60)
	No	N/A	No	(45)
β-thalassemia	*In vitro*	Yes—reduced hematopoietic potential and absence of erythrocyte colonies	No	(71)
	*In vitro*	Yes—impaired erythrocyte colony formation	Yes–gene correction (generation of isogenic control)	(78)
Colorectal cancer (CRC)	*In vitro*	Yes—increased WNT signaling and enhanced proliferation of colonic epithelial cells	No	(43)
Diamond-Blackfan anemia (DBA)	*In vitro*	Yes—defective erythropoiesis	No	(44, 47)
Familial platelet disorder with acute myeloid leukemia (FDP/AML)	*In vitro*	Yes—defective hematopoiesis and impaired erythrocyte and megakaryocyte differentiation	No	(38, 42, 52, 64)
Fanconi anemia (FA)	*In vitro*	No—robust multilineage hematopoietic differentiation potential with a non-significant reduction in erythroid and myeloid cell colonies	No (but viral gene complementation before reprogramming)	(62)
	*In vitro*	Yes—reduced clonogenic potential and increased apoptosis of hematopoietic progenitors	No	(75)
	*In vitro*	Yes—defective hemangiogenic progenitors resulting in ineffecient differentiation to hematopoietic and endothelial lineages	No	(68)
Glanzmann thrombasthenia (GT)	*In vitro*	Yes—absence of membrane expression of integrin αIIbβ3, reduction of platelet activation marker binding, impaired adherence to fibrinogen and defective platelet aggregation		(51, 63)
Juvenile myelomonocytic leukemia (JMML)	*In vitro*	Yes—enhanced production of myeloid cells with increased proliferative capacity and GM-CSF hypersensitivity	No	(46)
Juvenile myelomonocytic leukemia/Noonan Syndrome (JMML/NS)	*In vitro*	Yes—enhanced production of myeloid cells with increased proliferative capacity and GM-CSF hypersensitivity	No	(61)
Li-Fraumeni Syndrome (LFS)	*In vitro, in ovo* & *in vivo*	Yes—osteosarcoma features including aberrant osteoblast differentiation and tumorgenicity, and involvement of *H19*	no	(58)
	*In vitro, in ovo* & *in vivo*	Yes—osteosarcoma features including aberrant osteoblast differentiation and tumorgenicity, and paracrine and autocrine role of *SFRP2* in osteosarcomagenesis	yes—introduction of P53 mutations	(54)
Lymphangioleiomyomatosis (LAM)	*In vitro*	Yes—increased mTORC1 activation, abnormal autophagy and LAM-associate biomarker expression in smooth muscle cells	No	(53)
Multiple endocrine neoplasia type 2A (MEN2A)	No	N/A	Yes—mutation correction (generation of isogenic control)	(48, 79)
Myelodysplastic syndrome (MDS)	*In vitro*	Yes—drastically reduced hematopoietic differentiation potential and myeloid clonogenicity; increased cell death during *in vitro* differentiation	Yes—introduction of disease associated chr7q deletion	(55)
	*In vitro*	Yes—mild perturbation of hematopoietic differentiation with morphologic dysplasia	Yes—introduction and correction of disease associated *SRSF2* P95L mutation	(40)
	*In vitro*	Yes—reduced ability to generate granulocyte-erythrocyte-macrophage-megakaryocyte and erythrocyte colonies *in vitro*	Yes—introduction of disease associated mutations	(56)
Myelodysplastic syndrome with acute myeloid leukemia (MDS/AML)	*In vitro* & *in vivo*	Yes—reduced ability to generate granulocyte-erythrocyte-macrophage-megakaryocyte and erythrocyte colonies *in vitro*, and robust leukemia development *in vivo*	Yes—introduction of disease associated mutations	(56)
Myeloproliferative neoplasm (MPN)—Chronic myeloid leukemia (CML)	*In vitro*	Yes—reduced hematopoietic differentiation	No	(39)
	*In vitro* & *in vivo*	Yes—CML-iPSC–derived hematopoietic cells were sensitive to imatinib	No	(57)
	No	N/A	No	(50)
Myeloproliferative neoplasm (MPN)—Essential thrombocythenia (ET)	*In vitro*	Yes—increased megakaryopoiesis	No	(69)
Myeloproliferative neoplasm (MPN)—Primary and secondary myelofibrosis (PMF/SMF)	*In vitro*	Yes—increased expression of MF-associated IL-8 in megakaryocytes	No	(49)
Myeloproliferative neoplasm (MPN)—Polycythemia vera (PV)	*In vitro*	Yes—increased erythropoiesis & PV patient similar gene expression	No	(74)
	*In vitro*	Yes—increased megakaryopoiesis and erythropoiesis; increased sensitivity to EPO and TPO	No	(65)
	*In vitro*	Yes— EPO-independent erythropoiesis	No	(73)
Pancreatic ductal adenocarcinoma (PDAC)	*In vivo*	Yes—development of pancreatic intraepithelial neoplasm (PanIN) precursors to PDAC, which subsequently progressed further to the invasive stage	No	(33)
Shwachman-Diamond syndrome (SDS)	*In vitro*	Yes—impaired exocrine pancreatic and hematopoietic differentiation with reduced myeloid cell generation *in vitro*, increased apoptosis, and elevated protease activity	No	(70)
Sickle cell disease (SCD)	No	N/A	Yes–mutation correction	(76)
	No	N/A	Yes–mutation correction	(66)
	No	N/A	Yes–mutation correction	(67)
Trisomy 21	*In vitro*	Yes—increased numbers of CD43^+^CD235^+^ erythroid-megakaryocyte progenitors, and erythrocyte, granulocyte, macrophage, and megakaryocyte colonies	No	(77)

## Advantages of iPSCs

One of the main advantages of the iPSC technology is that hematological disease-associated and malignant lesions can be studied with human cells and in the genomic context of the patient. This is of considerable importance given that certain non-human models are not reflective of the human condition. An example is familial platelet disorder with a tendency to develop acute myeloid leukemia (FPD/AML) that is caused by inherited monoallelic mutations in *RUNX1* (80). FPD/AML presents with mild to moderate thrombocytopenia and bleeding due to impaired proplatelet formation, platelet activation defects, abnormal megakaryocyte differentiation and polyploidization, and a predisposition to develop AML (81). Neither, mouse nor zebrafish models of *RUNX1* mutations do develop a bleeding disorder or leukemia. In contrast, FDP/AML-iPSC derived “early wave” and “second wave” hematopoietic stem/progenitor cells showed aberrant hematopoiesis as occurs in FDP/AML patients (38, 42, 52, 64). Additionally, a person's genomic background greatly influences disease/cancer severity and progression as well as therapeutic response. Second, iPSCs provide a self-renewable, cryopreservable source of cells that are scalable to fulfill any need in cell numbers for cellular, biochemical, molecular, and other downstream applications. Third, with the appropriate cues and protocols iPSCs can be differentiated *in vitro* to many, in the future hopefully all cell types present in the body, enabling the study of multi-cell type affected diseases/cancers with one patient iPSC source. As an example, Tulpule et al. were able to show that Shwachman-Diamond syndrome (SDS)-iPSCs were impaired in both exocrine pancreatic and hematopoietic differentiation with reduced myeloid cell generation *in vitro*, increased apoptosis, and elevated protease activity recapitulating SDS patient phenotypes (70). Forth when the somatic cells used to generate iPSCs are isolated from primary hematological diseases/cancers or metastatic tumor specimens of non-germ line malignancies through biopsy, a bone marrow aspirate or blood sampling, normal cells will be inadvertently co-isolated along the malignant cells. Thus, the same reprogramming event can simultaneously generate paired malignant and normal iPSCs that share the same genetic background with exception of the disease-associated/cancerous lesion(s) in the malignant iPSCs. Distinguishing the normal iPSCs from the disease/cancer iPSCs has to be done retrospectively through genetic analysis (33, 55, 73). Alternatively, isogenic normal iPSCs can be established independently through a separate reprogramming experiment with somatic cells obtained from a non-malignant area adjacent to the tumor, a biopsy from an unaffected tissue such as the skin or from blood in the case of non-hematological disorders or cancers (33, 82). Another advantage of the iPSC technology is that reprogramming of malignant cells might establish iPSCs that represent various stages of disease progression, as cancers are often associated with serial accumulation of specific malignant mutations/lesions. Papapetrou et al. elegantly demonstrated this by using bone marrow or peripheral blood from four patients in different risk categories of myelodysplastic syndrome (MDS) or MDS/AML (56). They were successful in generating a library of iPSC lines that represents various disease stages including normal/healthy, preleukemia, low-risk MDS, high-risk MDS, and MDS/AML. The derived iPSC lines carried the respective gene mutations and chromosomal abnormalities found in the patients' bone marrow or peripheral blood cells used for reprogramming. Moreover, hematopoietic differentiation of these iPSC lines representing the various disease stages captured corresponding cellular phenotypes of graded severity and disease specificity.

## Limitations and challenges of iPSC modeling of human malignancies

Modeling hematological diseases and cancers with patient-specific iPSCs could face various hurdles due to technical, genomic stability and epigenome resetting challenges. It has been reported that some cancer cells are refractory to reprogramming (83, 84). This can have several reasons. For one, certain cancer cells and cells representing diverse stages may be difficult or even impossible to obtain and maintain for reprogramming purposes. Second, hematological diseases and cancers are often heterogeneous in nature, and reprogramming may preferentially select for cells with certain mutations and chromosomal aberrations and not others. Thus, the possibility exists that the panel of iPSC lines generated might not represent the entire heterogeneous composition of the patients' malignancy. Third, some cancer-associated mutations or genetic lesions might interfere with the reprogramming process itself or prevent maintenance of the pluripotent state. Fourth, even if iPSCs from patients with certain genetic lesions could be established, the specific lesions may render the cell genomically unstable. This will lead to acquisition of additional mutations and genomic abnormalities, which no longer reflect the cancer's genomic footprint and make the cells useless for proper disease modeling. Examples of unsuccessful reprogramming include the inability to establish iPSC lines from highly purified leukemic blast cells from patients with cytogenetically different subtypes of B cell-ALL (B-ALL) (84), and form Fanconi anemia (FA)-fibroblasts in one case (83). It is noteworthy to mention that FA-iPSC lines have been successfully generated (62, 68, 75). However, the FA pathway facilitates efficient reprogramming (62) and FA cells are genomically instable and predisposed to apoptosis (85). The latter is reflected by the observation of Yung et al. who showed that their FA-iPSC lines acquired significant additional abnormalities (hyperploidy) (75). The success in generating FA-iPSC likely might be dependent on the reprogramming condition—hypoxia appears better than normoxia (62) -, which FA-associated gene (fifteen genes constitute the FA complementation group) is mutated or even the kind of mutation. The derivation of AML-iPSCs, although successful for three AML patients with rearrangements in *KMT2A*/*MLL* (41, 59), has failed for AMLs with different mutations or lesions as well as *KMT2A*/*MLL* leukemic aberrations (41, 59). Stanford et al. also reported that *TSC2*-deficiency represents a barrier to reprogramming (53), while *TSC2*-happloinsufficient allowed iPSC generation with *TSC2*^+/−^-iPSC-derived smooth muscle cells recapitulating Lymphangioleiomyomatosis (LAM) features including increased mTORC1 activation, abnormal autophagy and LAM-associate biomarker expression (53).

Another possible limitation is the inability to derive cells of a defined cell type and developmental stage characteristic of the malignancy from iPSCs. Although protocols for generation of many general cell types have been established, the signaling cues and *in vitro* differentiation protocols for certain specialized cells, and developmental and maturation staged are still not fully understood. This is further complicated by the fact that differentiation and maturation efficiency is never 100% and, in most cases, the differentiation and maturation stage of a given cell within a population cannot easily be discriminated, thus, potentially hampering the correlation of disease phenotypes with the cellular phenotypes present in the culture. This issue could be resolved by introduction of stage-specific reporter genes via genome editing or by detailed stepwise characterization of the stages of differentiation and maturation in order to identify the exact stage at which the disease phenotype manifests. Additionally, the constant technological advances in single cell analyses at the cellular and molecular level will greatly improve disease modeling and mechanistic studies.

Cell reprogramming is associated with resetting of the starting cell's epigenetic landscape to that of a pluripotent stem cell. This resetting might eliminate characteristic features of the disease/cancer cell phenotype that might not be recreated upon differentiation, thus producing a significant difference between the disease/cancer iPSC model and the original disease/cancer cell. Here, it is worth bringing forth the theory that the initial oncogenic insult to the cancer-initiating cell might (re)program the epigenome toward a specific cancer cell fate (86). This potentially important aspect of malignancy could well be lost in iPSCs as reprogramming to iPSCs is accompanied by genome-wide epigenetic resetting (see *Epigenome, Cancer, and iPSCs*). Additionally, if one agrees with Sánchez-García's tumor stem cell reprogramming viewpoint that cancer cell properties can reemerge upon differentiation and that this property is to a fixed, uni-differentiated cell fate then this may not reemerge in an iPSC model due to the fact that iPSCs by definition possess pluripotent differentiation ability. On the other hand, such a resetting might be looked at favorably in certain diseases/cancers of “pure” epigenetic origin for which one could envision of using cells differentiated from these epigenetically reset iPSCs as a regenerative therapy.

Last but not least, modeling systemic processes *in vitro* is a challenge, as generally iPSC are maintained isolated as functionally autonomous entities in two-dimensional culture systems and not physiological integrated within the disease/tumor microenvironment. Recent progress and use of tissue engineering, three-dimensional organoids, MPS and *in vivo* xenografts offers a window to more sophisticated modeling that enables incorporation of malignant cells with cellular and extracellular components of the disease/tumor microenvironment, nutrient supply, and mimicking of blood/lymph flow thus attempting to recapitulate the *in vivo* architecture and physiological condition in which the malignant cells reside and grow.

## Epigenome, cancer, and iPSCs

Hematological diseases and cancers are profoundly influenced by changes in the epigenome and associated with a specific epigenetic profile. Since reprogramming to pluripotency is achieved through a stepwise resetting of the epigenetic landscape of the starting cell to that of a self-renewing, pluripotent iPSC (87), it is foreseeable that under certain circumstances this could have a negative impact on specific disease/cancer iPSC-based models. For example, iPSCs derived from non-small cell lung cancer (NSCLC) cell lines reset the NSCLC-associated transcriptional and methylation pattern of associated oncogenes and tumor suppressors (31). Similarly, Zhang et al. showed that reprogramming of sarcoma cell lines with complex, abnormal karyotypes to iPSCs resets the sarcoma transcriptional and epigenetic pattern and that the derived iPSCs gained self-renewal and multi-lineage differentiation potential (30). Neither of these studies examined whether the cancer-associated epigenetic profile reminiscent of the original cancer cell could be reestablished upon differentiation. Comparably, iPSCs generated from patients with AML carrying *MLL* rearrangements retained the leukemic mutations but also reset leukemic DNA methylation and gene expression patterns (41). However, leukemic DNA methylation and gene expression profiles reemerged in AML-iPSC-derived hematopoietic cells. Similarly, human glioblastoma-derived iPSCs remain highly malignant after differentiation into neural progenitors and pancreatic ductal adenocarcinoma (PDAC)-iPSCs establish secondary pancreatic-cancer in patient-derived xenografts (see also below) (32, 33). These examples suggest that cancer cell properties, albeit reset in iPSCs, can reemerge upon differentiation to the appropriate cancer cell type.

Remarkably, a recent report showed that the cellular context could significantly impact on the genetic information and behavior of malignant cells (88). Hashimoto et al. reprogrammed mouse colon tumor cells with loss of *Apc*. The reprogrammed tumor cells, *Apc*-iPSCs, displayed iPSC-like morphology and gene expression but lacked pluripotency and showed a trophectoderm-differentiation bias. Surprisingly, the majority of genes affected by the Apc mutation in *Apc*-iPSCs were different than those affected in the colon. Genetic *Apc*-rescue coupled with a subsequent deletion strategy revealed neoplastic growth specific to intestinal cells but not other cell types *in vivo*. It is noteworthy though that the majority of *Apc*-iPSC-derived colonic legions remained in a pretumoral microadenoma stage and did not develop into full blown macroscopic colon tumors. These findings imply that disease cell properties and biological consequences of tumor-causing mutations are strongly depending on the cellular context and underscore that epigenetic regulation, which is critical for cell fate determination and fixing the malignant cell state in cancer (see also our discussion of this issue in *Limitations and challenges of iPSC modeling of human malignancies*), exerts great influence on disease development and progression.

## Genome editing

Genetic modification of human pluripotent stem cells through conventional homologous recombination is hampered by extremely rare recombination events (89). Recent advances in genome editing technologies (zinc finger nucleases (ZFN), transcription activator-like effector nucleases (TALENS) and Clustered Regularly Interspaced Short Palindromic Repeats (CRISPR) with the Cas9 nuclease) that enable precise genetic modifications at the single nucleotide level efficiently are gaining wide use in iPSC disease modeling, including the investigation of hematological diseases and cancers (90–92). Genome editing can be used to correct or introduce disease-associated mutations, individually or in combinations, into patient-specific iPSCs or normal iPSCs, respectively, thus enabling systemic interrogation of gene function and disease development (89, 93). In both correction or introduction of mutation cases, iPSCs will be generated that bear the same genomic background and only differ in the specific genetic alteration, thus, providing ideal, isogenic iPSC pairs for comparative analysis. Genome editing through non-homologous end-joining will generate frame-shift mutations through introduction of small, random nucleotide insertions or deletions (indels) and, hence, is well suited for monoallelic or biallelic inactivation of haploinsufficient or classical tumor-suppressor genes. On the other hand, homology-directed repair (HDR) utilizing co-delivery of homologous donor DNA template to guide the homologous recombination-mediated repair process, will generate precise modifications and, thus, can be used to study point mutations in disease/cancer-associate genes or associated regulatory regions. For example, using ZFN or TALEN-based HDR, several groups succeeded in correcting of the causative, single-nucleotide mutations in *HBB* in sickle cell disease (SCD) and β-thalassemia iPSC lines (66, 67, 76, 78). Ma et al. showed that two distinct β-thalassemia major patient-corrected iPSC lines showed increased erythrocyte colony formation of hematopoietic progenitors compared to their isogenic, mutant iPSCs (78). Papapetrou et al. have conducted some of the most elegant gene editing for hematological malignancy modeling (56). Using correction or introduction of mutations via CRISPR/Cas9 in combination with patient-specific diseased or normal iPSCs, they modeled various disease progression stages ranging from normal/healthy, preleukemic, low-risk MDS, high-risk MDS to MDS/AML (56) as well as the contribution of the splicing factor SRSF2p.P95L mutant to MDS alone or in the context of MDS with del(7q) (40).

Genome editing systems can also be used to introduce or revert large-scale genetic lesions often associated with specific malignancies, including chromosomal deletions, inversions and translocations (55, 94–97). Brunet et al. used ZFNs and TALENs in human cells, including embryonic stem cell-derived mesenchymal precursors to generate *t*_(11;22)_(q24;q12) *EWSR1-FLI1* fusion and *t*_(2;5)_(p23;q35) *NMP1-ALK* fusion genomic translocations associated with Ewing sarcoma and anaplastic large cell lymphoma, respectively, or to revert the *t*_(2;5)_(p23;q35) *NMP1-ALK* translocation (95). Torres-Ruiz et al. using CRISPR/Cas9 successfully recreated the *t*_(11;22)_(q24;q12) *EWSR1-FLI1* fusion translocation in iPSCs (97). Using the adeno-associated vector-mediated gene targeting of an HSV-tk transgene approach, Papapetrou et al. generated various deletions of chromosome 7q that let them to identify an approximately 20 Mb region spanning 7q32.3-7q36.1 as the critical region in del(7q)-associated MDS (55). We together with our colleagues and the late Ihor R. Lemischka previously generated iPSCs from a Li-Fraumeni syndrome (LFS) family to investigate the oncogenic role of mutant TP53 in the development of LFS-osteosarcoma (58). In a follow up-study we identified SFRP2 as an autocrine and paracrine factor involved in P53 mutation-mediated osteosarcomagenesis. Using genome-editing we confirmed a correlation between various P53 mutations and increased SFRP2 expression in iPSC and embryonic stem cell derived osteoblasts (54) and Kim et al. (under review).

## Integration of iPSCs with tissue engineering, three-dimensional organoids and microphysiological systems

Diseases and cancers do not occur in a two-dimensional vacuum of malignant cells in culture but rather involve complex interactions and communication with neighboring cells and the microenvironment. Cells in the niche and the extracellular matrix provide anchor, biomechanical support and spatiotemporally regulated biochemical signals and nutrients needed for disease initiation, progression and survival. The use of tissue engineering, three-dimensional organoids and MPS attempts to more faithfully mimic the *in vivo* cellular milieu, architectural structure, spatial organization and physiological parameters than two-dimensional culture systems ever could. Integration of directed differentiation of iPSCs with tissue engineering, organoid cultures MPS are being developed for many complex tissues such as the heart, liver, kidney, intestine, eye, and brain (98, 99).

Organoids derived from primary resected tumors or biopsies are hailed to create opportunities to build large biobanks with relevant patient material for cancer research, drug evaluation and therapy development (100–109). With the goal of modeling human diseases of the large intestine, Chen et al. developed an efficient colonic organoid (CO) strategy using embryonic stem cells and iPSCs (43). Through a stepwise differentiation protocol following progressive normal development of definitive endoderm to hindgut endoderm to subsequently COs, using patient-specific colorectal cancer familial adenomatous polyposis (FAP)-iPSCs that carry a germline nonsense mutation in *APC* causing early termination of translation, they were able to demonstrate enhanced WNT signaling and increased epithelial cell proliferation. Additionally, they used these FAP-iPSC COs as a platform for testing drugs (see *iPSCs in drug development & pharmacology*).

As discussed in *iPSCs in xenograft models*, Zaret et al. modeled PDAC development using PDAC-iPSCs in combination with *in vivo* transplantation (33). In order to establish an *in vitro* model of early stage human pancreatic cancer, they harvest the PanIN structures from the developing PDAC-iPSC-derived teratomas and set up organoid cultures. The formed organoids retained PDAC-associated marker expression and served as a platform for biomarker identification.

MPS, also known as microfluidic organ-on-a-chip, offer a precise means to integrate cells, including iPSC-derived cell types and 3-dimensional constructs or organoids, into an *in vitro* dynamic system that further incorporates vascular flow and micro-biofabrication that mimics the systematic architectural and spatial compositions and interactions among different cell-types, tissues and organs in the body. Use of MPS in cancer research is gaining traction to investigate complex cancer, growth, tumor-niche interactions, metastatic invasion, and drug delivery, efficacy and toxicity (13–20). However, the incorporation of iPSCs or derived progenies into MPS is just beginning (110–113). Advances in generating higher-order MPS that are able to link individual systems into a physiome- or body-on-a-chip (114, 115) coupled with inline detectors and fluorescent reporters (116–119) will enable dynamic, real-time interrogation of cellular, molecular, and biomechanical parameters of disease pathogenesis (initiation and progression) and drug responses.

## iPSCs in xenograft models

Patient-derived xenografts (PDXs) have become a prominent model system as they are presumed to more faithfully capture the cellular, molecular and physiological characteristics of primary and metastatic malignancies (120, 121). Additionally, PDX-models are gaining attraction in such field as biomarker identification, drug development and assessment of drug responses (122).

Transplantation of iPSCs or derived cells into appropriate animal models can provide a more physiological, three-dimensional *in vivo* environment and, hence, expand their experimental utility. PDAC has a very poor prognosis and until the elegant study by Zaret et al. lacked a human cell model of early disease progression (33). Subcutaneous, injection of iPSCs into immunocompromised mice is a process used to assess the pluripotency of iPSCs through the formation of teratomas. When Zaret et al. injected PDAC-iPSCs, ductal structures formed within the developing teratomas that had a more prominent architectural organization compared to controls. Detailed cellular and molecular characterization of these structures let to the conclusion that they resembled PanIN-stage like structures that eventually further progressed to an invasive PDAC stage.

Majeti et al. established an AML model based on iPSCs generated form patients with rearrangements of the *KMT2A*/*MLL* locus (41). Using intravenous or orthotopic transplantation into immunocompromised mice to evaluate leukemia formation in *vivo* they found that the ability to give rise to leukemia *in vivo* is dependent on transplantation of AML-iPSC-derived hematopoietic cells as AML-iPSCs lacked leukemic potential. Additionally, despite retaining the leukemic-driver mutations, AML-iPSCs reset the leukemic DNA methylation and gene expression patterns. Surprisingly, hematopoietic differentiation of these AML-iPSCs and leukemia formation was sufficient to reestablish the leukemic DNA methylation and gene expression profile strongly suggesting that the genetic mutations/rearrangements of the *KMT2A*/*MLL* locus in AML-iPSCs reactivate a leukemic program in the context of hematopoietic cells (41).

It was recently reported that copy number alterations recurrently observed in primary human tumors gradually disappeared in PDXs, suggesting that events undergoing positive selection in humans can become dispensable during propagation in mice (123). In light of this observation and its critical implications for PDX-based disease/cancer modeling, cytogenetic analyses of PDX-donor cells after *in vivo* transplantation and propagation appears important in order to know whether the attempted PDX-model accurately retains the genetic lesions present in the original malignant cells or if they evolve, and if they evolve whether the evolution is specific to the patient or the host.

## iPSCs in drug development and pharmacology

The cost of drug development from discovery, through clinical trials to approval and marketing is in excess of $2.6 billion (124). As costly as clinical trials are, drug failures are key contributors to development costs. Induced PSCs and derived cells are gaining attraction and are being more widely used in translational-research settings, including discovery and validation of biomarkers and therapeutic targets, compound screening for drug discovery and drug repurposing, and preclinical drug susceptibility, efficacy and toxicity studies (33, 39, 41, 43, 57, 65, 72, 73, 110, 125–131). Of particular usefulness is that many different cell type, including cardiomyocytes, hepatocytes, neurons, and hematopoietic cells, can readily be generated from a diverse set (age, gender, race/ethnicity) of iPSCs from healthy individuals or patients with a given disease/cancer. This has been exemplified in the use of iPSCs in drug toxicity screening. Therapeutically effective drugs can cause serious unintended adverse events that limit or even prohibit their use. Several groups have used iPSC-derived cardiomyocytes to model and investigate anticancer drug-induced cardiotoxicity (132–137). In one case, cardiomyocytes generated from iPSCs from breast cancer patients were able to recapitulate patient-specific doxorubicin-induced cardiotoxicity at the cellular level (134). Another application is the evaluation of drug susceptibility and variable responses of phenotypic distinct cell populations, cancer subclones or patients (39, 41, 57, 65, 72, 73). Primary or acquired-drug resistance is a serious clinical problem. Induced PSCs derived either from drug-sensitive and drug-resistance patients or from cells of the same patients at the drug-sensitive and drug-resistant stage and iPSC derived cells might help decipher the mechanisms underlying drug-resistance. Examples along this line are from Bedel et al. (39) and Kumano et al. (57). They derived iPSC lines from CML patients that carry the abnormal Philadelphia chromosome that resulted from a translocation between chromosome 22 and 9 leading to the fused, oncogenic BCR-ABL tyrosine kinase. While both groups reported that the generated CML-iPSC lines were resistant to the tyrosine kinase inhibitor imatinib, which is used to treat CML patients, Bedel *et al*. (39) found that CD34^+^ hematopoietic progenitors obtained from their patient's CML-iPSCs were partially sensitive to imatinib and Kumano et al. (57) found imatinib-sensitivity in CML-iPSC derived CD34^−^ hematopoietic cells but not CD34^+^ hematopoietic progenitors, which recapitulated the pathophysiological feature of initial CML of that patient. In depth molecular characterization at the epigenome, transcriptome and proteome level will be necessary to discover the signaling networks responsible for the observed behavior. Induced PSCs and derived cells also present an opportunity for phenotypic drug testing and screening. This can be especially attractive for diseases with no previously characterized targets or drug treatment strategies. However, such phenotypic drug testing and screening requires the ability to identify cellular phenotypes or functional properties, such as proliferation, apoptosis, activation of a specific signaling pathway, a distinct metabolic profile that correlate with patient phenotypes and responses and thus can serve as surrogate readouts of therapeutic effectiveness (43, 110, 117, 130, 138). Undoubtedly, the next stage in drug discovery and pharmacological testing will expand on the integration of iPSC-based model systems with three-dimensional organoids and MPS (43, 110).

## Concluding remarks

iPSC technology started a new, exciting era in biomedicine. The ease by which patient-specific iPSCs from various primary or metastatic somatic tissues and blood of patients with hematological diseases and cancers can be derived provides a self-renewable, scalable and cryopreservable source of cells with the patient's genetic background. iPSCs are readily enable to genome-editing in order to either correct or introduce known or suspected disease-associated mutations. This novel tool enables attempts to successfully recapitulate various pathological disease states and features associated with malignancies in a patient-specific context. Integration of iPSC-based disease and cancer models with advanced, bioengineered physiological systems, *in vivo* PDX models, automated high-throughput-screening tools and next-generation omics approaches will lead to a greater mechanistic understanding of disease/cancer, the relationship between malignant cells and their microenvironment, and drug responses. Undoubtedly, iPSC technology is revolutionizing the way we approach disease modeling, preclinical cancer research, drug development and precision medicine.

## Author contributions

All authors listed have made a substantial, direct and intellectual contribution to the work, and approved it for publication.

### Conflict of interest statement

The authors declare that the research was conducted in the absence of any commercial or financial relationships that could be construed as a potential conflict of interest.
